# Diffuse-Type Tenosynovial Giant Cell Tumor of the Knee: Clinical Course After Anterior Open Synovectomy

**DOI:** 10.3390/curroncol32060342

**Published:** 2025-06-11

**Authors:** Alessandro Bruschi, Eric Staals, Andrea Sambri, Luca Cevolani, Marco Gambarotti, Alberto Righi, Michele Fiore, Eleonora Villari, Stefano Pasini, Maria Giulia Pirini, Massimiliano De Paolis, Davide Maria Donati

**Affiliations:** 1 Orthopedic and Traumatology Unit, IRCCS Azienda Ospedaliero-Universitaria di Bologna, 40138 Bologna, Italy; alessandro.bruschi@aosp.bo.it (A.B.); michele.fiore@aosp.bo.it (M.F.); eleonora.villari@ior.it (E.V.); massimiliano.depaolis@aosp.bo.it (M.D.P.); 2 Department of Medical and Surgical Sciences, Alma Mater Studiorum University of Bologna, 40138 Bologna, Italy; 3 Orthopaedic Oncology Unit, IRCCS Istituto Ortopedico Rizzoli, Via Pupilli 1, 40136 Bologna, Italy; eric.staals@ior.it (E.S.); luca.cevolani@ior.it (L.C.); davide.donati@ior.it (D.M.D.); 4 Department of Pathology, IRCCS Istituto Ortopedico Rizzoli, 40136 Bologna, Italy; m.gambarotti@ior.it (M.G.); a.righi@ior.it (A.R.); 5 Department of Biomedical and Neuromotor Sciences, University of Bologna, 40123 Bologna, Italy; 6 Spine Surgery Unit, IRCCS Istituto Ortopedico Rizzoli, 40136 Bologna, Italy; stefano.pasini@ior.it; 7 Pathology Unit, IRCCS Azienda Ospedaliero-Universitaria di Bologna, 40138 Bologna, Italy; mariagiulia.pirini@aosp.bo.it

**Keywords:** tenosynovial giant cell tumor, pigmented villonodular synovitis, range of motion, pain, swelling

## Abstract

Diffuse type tenosynovial giant cell tumor (D-TGCT) of the knee presents with stiffness, pain and swelling with surgery being the mainstay treatment. However, the literature lacks data on clinical course of range of motion, pain (ROM), and swelling after open synovectomy for D-TGCT. Therefore, this study aims to evaluate clinical course after open anterior synovectomy. A retrospective analysis was conducted on 214 patients treated for TGCT at our Institutions between 2010 and 2023. 51 patients with anterior knee D-TGCT who underwent open anterior synovectomy were included. Pre- and postoperative assessments included ROM, pain (VAS scale), and reported swelling. The mean knee flexion increased from 100° (SD 14.28) preoperatively to 131.8° (12.64) at 12 months post-surgery. Knee extension remained stable, ranging from 178.4° preoperatively to 179.2° at the final follow-up. Pain decreased from a mean of 5.0 (SD 2.8) preoperatively to 0.5 (SD 0.7) at 12 months. Swelling was initially reported in 90.4% of patients, with 95.74% of them showing improvement at six months, and 100% at 12 months. Open anterior synovectomy effectively improves knee function, pain, swelling in patients with anterior knee D-TGCT, although functional recovery may take up to 6–12 months.

## 1. Introduction

Tenosynovial giant cell tumors (TGCTs) is a group of rare, proliferative lesions that involve the synovium of joint, bursa and tendon sheath with a prevalence of 9.2 patient/million population [1].

Since 2013, the World Health Organization (WHO) distinguishes two types of TGCT: (a) single nodule (localized form: L-TGCT) and (b) multiple nodules (diffuse form: D-TGCT). Localized TGCT encompasses previously known giant cell tumor of tendon sheath, while the diffuse GCT encompasses the already-known nodular tenosynovitis and pigmented villonodular synovitis [2].

Diffuse TGCT typically affects large joints. The most frequently involved site is the knee, followed by the hip, ankle, elbow, and shoulder, while the spine is rarely involved. It usually affects a single joint; nonetheless, occurrence in multiple joints is described in the literature in children with other conditions such as vascular lesion, cherubism, extremity lymphedema, Noonan syndrome or jaw lesions [3].

Diffuse TGCT generally occurs in the third or fourth decade of life and is more predominant in women than men [2].

The most frequent symptoms are progressively worsening pain, swelling and decreased range of motion (ROM). The disease can progress to degenerative and destructive changes in the joint cartilage and bone with chronic edema leading to a variable degree of joint stiffness. The latter is often detectable during the clinical evaluation, while it is usually not possible to palpate a soft tissue mass due to the intra-articular localization [4,5].

To date, surgical excision is the mainstay treatment; however, for symptomatic patients with TGCT not amenable to improvement with surgery, systemic therapies that block the CSF-1/CSF-1R pathway are emerging as an alternative [6,7,8,9,10,11,12,13]. Nevertheless, if symptoms are not considerably disabling, quality of life is acceptable and radiographically joint damage is minimal, it is also possible to adopt a conservative approach to watchful waiting [12,14].

Depending on the amount of disease, synovectomy can be performed by open, arthroscopic, or even combining the two techniques, although a higher relapse rate has been reported after arthroscopic treatment [5,15,16,17,18]. In the case of massive joint involvement, extensive surgical debridement up to total knee arthroplasty is required [19,20]. The combination of the size and location of the lesion influence the surgical approach which can be anterior, posterior, or combined [21].

Due to the inflammatory state caused by the disease and by surgical procedure, patients can strive to promptly recover ROM, to reduce pain and swelling. However, in the current literature, studies on D-TGCT are limited regarding the recovery after open synovectomy and what should be expected in follow-up visits, in particular on the restoration of ROM, pain and swelling reduction [22].

Therefore, the aim of this study is to report clinical course of ROM, pain and swelling after open anterior synovectomy for knee D-TGCT.

## 2. Patients and Methods

### 2.1. Patient Selection

**Figure 1:**
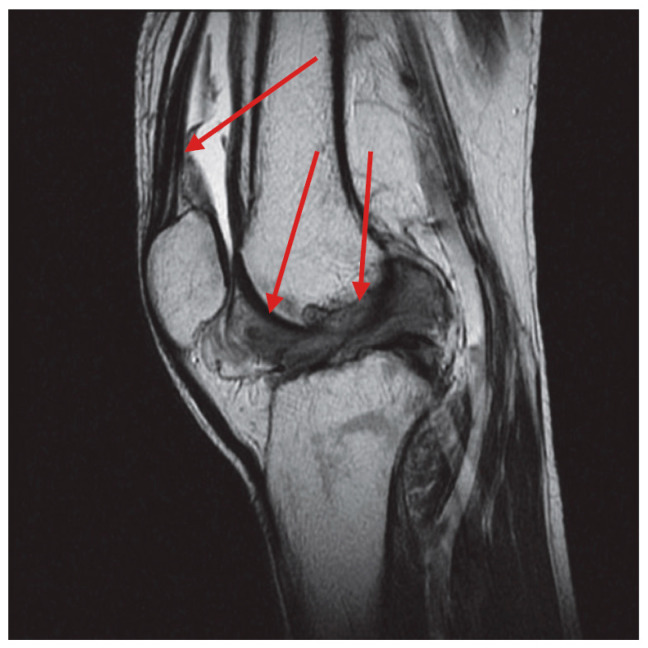
T2-weighted magnetic resonance sagittal scan of a D-TGCT. The location of disease is indicated by red arrows.

**Figure 2:**
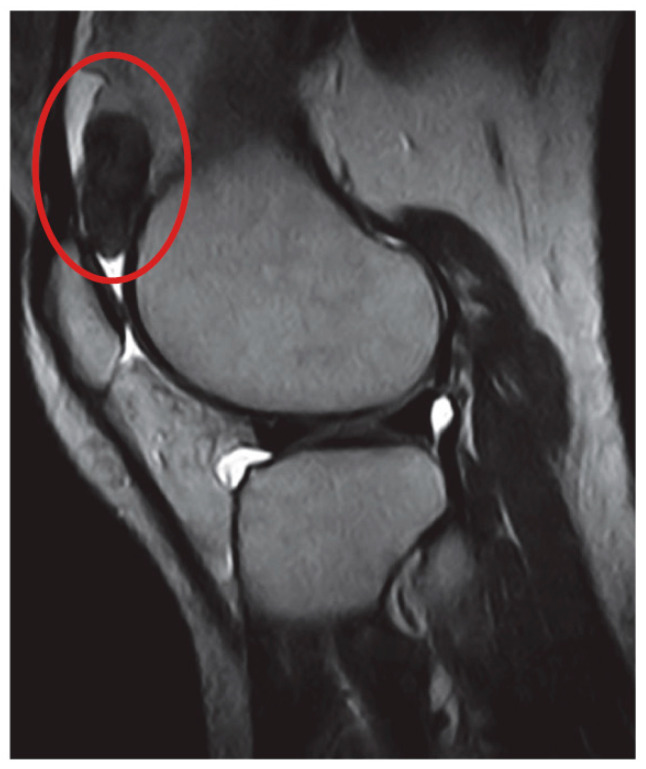
T2-weighted magnetic resonance sagittal scan of L-TGCT. The location of disease is indicated by a red circle.

We retrospectively reviewed 214 patients suffering from TGCT, treated at our Institutions between 1 January 2010 and 31 December 2023. The recruitment of patients was continuous during the study period. Magnetic resonance imaging (MRI) was used for diagnosis and to assess extension and localization of the disease; core needle biopsy with a 14G needle was performed in all patients when the MRI exam did not meet the radiological criteria for the diagnosis. As reported in the literature, we considered a diagnosis of D-TGCT in cases when one or multiple nodules were present in the synovial membrane of the articular cavity without distinct margins and diagnosis of L-TGCT diagnosis when a single well-defined nodule was present. (Figure 1 and Figure 2) [23].

**Figure 3:**
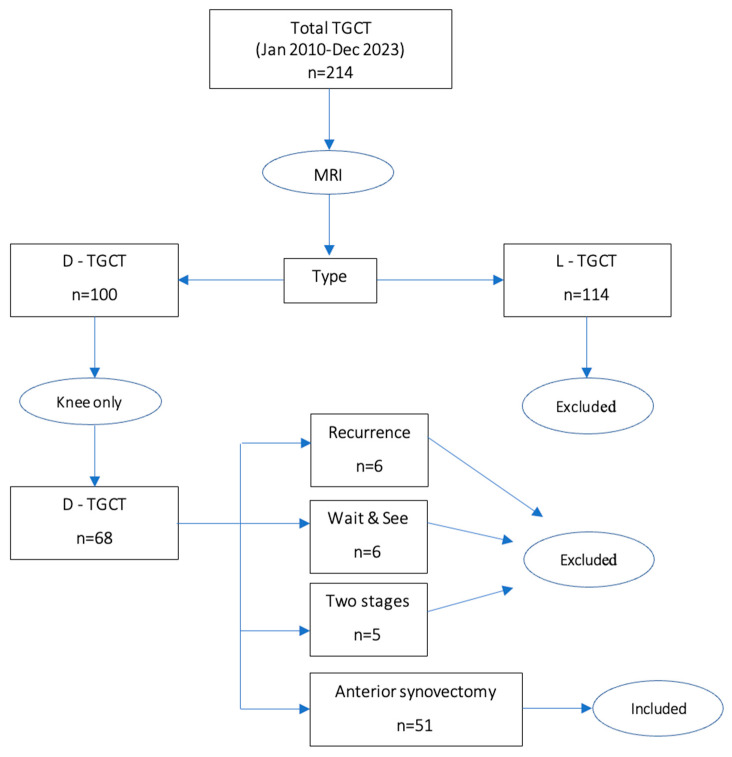
Patients selection.

The diagnosis was confirmed postoperatively in all patients by histological examination of the specimen. We excluded patients with localized-type TGCT (n = 114) and those with a FU shorter than 12 months. Of the 100 patients affected by D-TGCT we excluded the 32 patients with no knee involvement. All open anterior synovectomy were performed by orthopedic oncology surgeons with more than ten years of experience in treating this disease. No systemic therapy was administered. According to those criteria, 62 patients (36 female and 26 males, with an average age of 42.1 years, ranging from 23 to 64) treated for diffuse-type TGCT of the knee were included. We excluded 5 patients with disease also in the posterior compartment according to MRI results, who were treated in two stages through anterior and posterior approach. We also excluded 6 patients that were treated conservatively with watchful waiting because they refused surgery. Other 6 patients were excluded for recurrence of the disease to avoid biases due to recurrence in the postoperative course analysis (Figure 3).

Pre-operative symptoms were recorded. VAS scale was used to assess pain [24]. Swelling was assessed during physical examination using patellar tap test and comparing the affected knee with the contralateral.

ROM was evaluated independently as flexion (ROF-range of flexion) and extension (ROE-range of extension) with goniometers before surgery.

All patients underwent open synovectomy through a medial parapatellar approach.

After the surgery, all patients were re-evaluated ROF and ROE at 1, 3, 6 and 12 months and the course of pain and swelling was recorded at 6 and 12 months. All the assessments and treatments reported for this retrospective study were part of the normal clinical practice.

### 2.2. Open Anterior Synovectomy: Technique

With the patient in a supine position and a thigh tourniquet, a 12–14 cm median skin incision and medial peripatellar capsulotomy are performed. The anterior approach is carried out through an anteromedial parapatellar arthrotomy, ensuring the preservation of meniscal attachments and ligaments. Once the suprapatellar pouch is exposed, all tissue located deep to the quadriceps muscle and tendon, extending to the femoral periosteum, is excised en-bloc. Next, attention is directed to the undersurface of the patella, fat pad, distal femur, and proximal tibia. If the tumor is embedded within the fat pad, it must be completely removed. Any residual tumor within the medial or lateral gutter or beneath the menisci was excised using a standard or pituitary rongeur or curettes. Finally, the quadriceps tendon, subcutaneous tissue, and skin are sutured over a deep drain. Compressive bandage is used, and ice application is encouraged.

### 2.3. Statistical Analysis

All statistical analyses were performed using IBM SPSS Statistics version 29.0 (IBM Corp., Armonk, NY, USA). Continuous variables were reported as mean ± standard deviation (SD). The VAS scale was analyzed as an ordinal variable, with changes over time assessed using the Friedman test for repeated measures. A linear mixed-effects model was used to evaluate changes in ROF and ROE over time, accounting for individual patient variability.

## 3. Results

A total of 51 patients were included in the study—30 females and 21 males.

**Figure 4:**
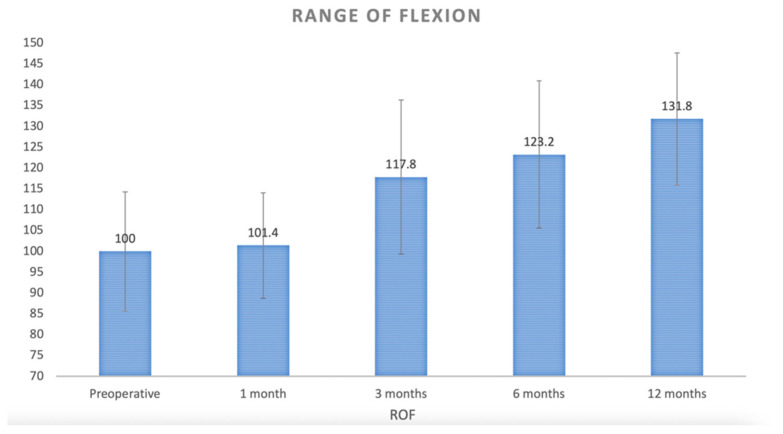
Clinical course of range of flexion (C.I: 95%; *p*: 0.031).

Before surgery, the mean ROF was 100.0° (SD 14.28) and became 101.4° (SD 12.64) at the initial follow-up one month after surgery. This improved to 117.8° (SD 18.47) at three months, 123.2° (SD 17.65) at six months, and 131.8° (SD 15.80) at twelve months follow-up (C.I: 95%; *p*: 0.031) (Figure 4).

**Figure 5:**
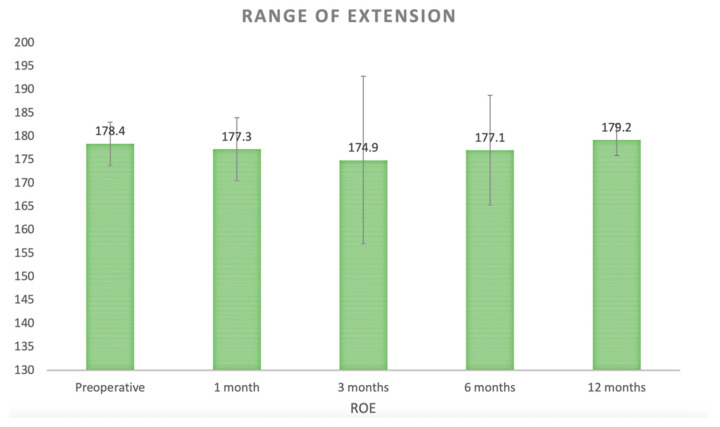
Clinical course of range of extension (C.I: 95%; *p*: 0.027).

The mean ROE pre-surgery was 178.4° (SD 4.590). At the first follow-up one month after surgery, the average extension was 177.2° (SD 6.73); it decreased to 174.9° (SD 17.93) at three months, and then to 177.1° (SD 11.71) at six months, to re-increase at twelve months with a measured mean range of 179.2° (SD 3.370) (C.I: 95%; *p*: 0.027) (Figure 5).

**Figure 6:**
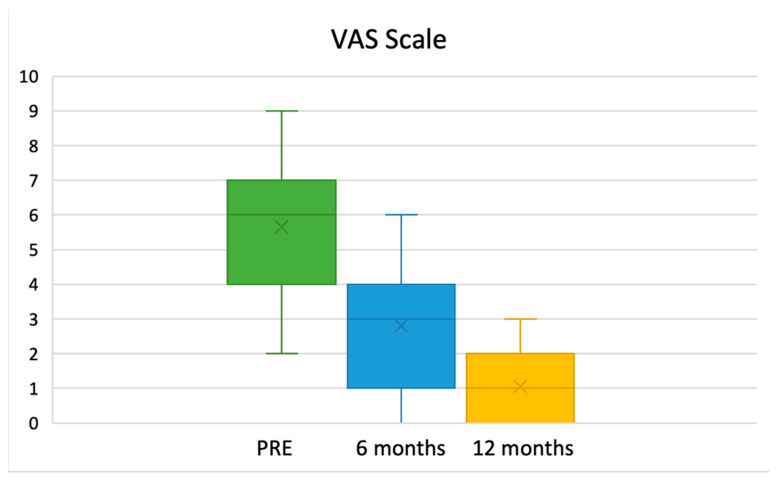
Course of pain based on VAS scale score (C.I: 95%; *p*: 0.021).

The mean VAS was 5.0 (SD 2.8) prior to surgery, and it improved to 3.0 (SD 4.2) six months after surgery and to 0.5 (SD 0.7) at the 12-month follow-up (C.I: 95%; *p*: 0.021) (Figure 6).

In 47 patients (90.4%), swelling was reported before surgery; 45 of them (95.7%) reported improvement at 6 months after surgery, and all patients reported further improvements at the 12-month follow-up.

## 4. Discussion

Our findings indicate that patients with anterior DGCT of the knee tend to exhibit an average ROF deficit of approximately 30 degrees compared to the full physiological flexion of about 130 degrees, preoperatively [25]. Following anterior synovectomy, there is only a minor improvement in mean knee flexion during the first postoperative month. This is likely due to the surgical insult and the associated postoperative swelling, which limit early recovery of motion [26]. However, in the subsequent months, progressive rehabilitation and physiotherapy facilitate a gradual improvement in flexion, ultimately reaching a mean postoperative flexion of 131.8 degrees in our cohort. Importantly, this return to near-normal knee flexion occurs over a prolonged period, typically between 6 and 12 months, postoperatively.

The delayed recovery of full flexion is primarily attributed to the significant inflammatory response within the anterior capsule of the knee joint, predominantly characterized by intra-articular effusion, which resorbs gradually. This prolonged inflammatory reaction is influenced by both the underlying predisposition of the knee to inflammation due to D-TGCT and the inherent surgical trauma associated with an anterior synovectomy.

Considering ROE, our results suggest that anterior synovitis has minimal impact on extension deficits, with values remaining close to the full physiological extension of about 180 degrees. A transient decrease in extension was observed at the three-month mark, likely due to joint swelling secondary to physiotherapy and the progressive return to daily and sporting activities, which typically occur around this time. Nonetheless, this effect appears to be temporary and does not affect long-term knee extension.

Regarding pain levels, our data indicate that pain is not a severely debilitating symptom in most patients. The average preoperative pain level was measured at 5.0 on the VAS scale. This gradually improved within the first six months to an average score of 3.0 and further decreased over the following six months, reaching a near-negligible mean value of 0.5 at one year, postoperatively.

Finally, all patients reported a subjective improvement in swelling following surgery confirmed by physical examination. Progressive reductions in joint effusion were observed within the first six months, with an important improvement in all cases at the one-year follow-up.

In a large cohort of patients Mastboom et al. [27] reported pain improvement in 59% of patients and swelling improvements in 72% of patients; however, these data are difficult to compare with our case series as joints other than knee were included and different surgical treatment have been performed aside of anterior open synovectomy. In general, several authors underline that stiffness, pain and swelling are the most impactful symptoms affecting quality of life [28,29,30,31,32,33]. Our data are similar to what reported by Nakahara et al. on 17 patients [34]. Similar results are reported by Liu et al. on 97 patients at 15 months, however in their series 78 patients were treated with adjuvant radiotherapy and with a combination of open and arthroscopic approach in the majority of cases [35]. Yao et al. reported similar functional results on 295 patients, however the results are to some extent difficult to compare as we included only anterior localizations treated with open synovectomy, while they included also posterior location and they used a multiportal arthroscopic approach followed in many cases by adjuvant radiotherapy [36]. The recurrence rate in this study (11.8%), was better than in other comparable studies [18,37]. Overall, our results suggest that anterior synovectomy, despite the inflammatory reaction secondary to surgery and wound repair, contributes to significant reduction in the joint effusion and synovial hypertrophy, thereby improving the overall knee function and patient performance and satisfaction.

Given the subjective component involved in data collection and interpretation, the limitation of these study is that we reported a descriptive analysis of the post operative course of anterior open synovectomy for PVNS, with no interobserver analysis of data collection and interpretation and no paired Student’s *t*-test for specific comparisons (e.g., comparing follow-up with preoperative data). Another limitation is that we presented the course of swelling without quantitative data, only indirectly relating it to the quantitative data on course of ROM and pain. Moreover, the duration of the follow-up is short, and a longer follow-up would be needed to assess the long-term result of open anterior synovectomy for D-TGCT.

## 5. Conclusions

In conclusion, anterior synovectomy for D-TGCT is associated with an initial period of limited functional improvement due to postoperative inflammation and swelling. However, knee extension is not impaired and knee flexion progressively improves, pain decreases as well as swelling, leading to favorable long-term outcomes. However, these results require a rehabilitation period of 6 months, postoperatively. Future studies should aim to optimize the rehabilitation protocols and evaluate strategies to mitigate the initial inflammatory response to shorten the recovery period.

## Data Availability

The data presented in this study are available on request from the corresponding author.
